# Tumor Suppressor Inactivation in the Pathogenesis of Adult T-Cell Leukemia

**DOI:** 10.1155/2015/183590

**Published:** 2015-06-10

**Authors:** Christophe Nicot

**Affiliations:** Department of Pathology and Laboratory Medicine, Center for Viral Oncology, University of Kansas Medical Center, 3901 Rainbow Boulevard, Kansas City, KS 66160, USA

## Abstract

Tumor suppressor functions are essential to control cellular proliferation, to activate the apoptosis or senescence pathway to eliminate unwanted cells, to link DNA damage signals to cell cycle arrest checkpoints, to activate appropriate DNA repair pathways, and to prevent the loss of adhesion to inhibit initiation of metastases. Therefore, tumor suppressor genes are indispensable to maintaining genetic and genomic integrity. Consequently, inactivation of tumor suppressors by somatic mutations or epigenetic mechanisms is frequently associated with tumor initiation and development. In contrast, reactivation of tumor suppressor functions can effectively reverse the transformed phenotype and lead to cell cycle arrest or death of cancerous cells and be used as a therapeutic strategy. Adult T-cell leukemia/lymphoma (ATLL) is an aggressive lymphoproliferative disease associated with infection of CD4 T cells by the Human T-cell Leukemia Virus Type 1 (HTLV-I). HTLV-I-associated T-cell transformation is the result of a multistep oncogenic process in which the virus initially induces chronic T-cell proliferation and alters cellular pathways resulting in the accumulation of genetic defects and the deregulated growth of virally infected cells. This review will focus on the current knowledge of the genetic and epigenetic mechanisms regulating the inactivation of tumor suppressors in the pathogenesis of HTLV-I.

## 1. Introduction

The first description of HTLV-I came after the discovery of the human T-cell growth factor (interleukin-2; IL-2), allowing long-term* in vitro* culture of T cells and the establishment of T-cell lines from a patient with a cutaneous T-cell lymphoma [1–3]. Afterward, this virus was identified as the etiological agent of ATLL and the terminology HTLV-I was adopted. HTLV-I is transmitted through sexual contacts and contaminated blood and from mother to child by breast-feeding [4]. HTLV-I is mainly found in endemic areas such as Japan, Africa, South America, the Caribbean basin, southern parts of North America, and Eastern Europe [5]. The diversity in clinical presentation and prognosis of patients with ATLL has led to its classification into distinct subtypes referred to as smoldering, chronic, and acute or lymphoma type [6, 7]. In patients circulating atypical multinucleated lymphocytes termed “flower cells” are considered pathognomonic of ATLL. Tumor ATLL cells are of clonal origin and usually carry a single copy of integrated virus [8, 9]. The fact that the different clinical forms of ATLL have distinct genomic alterations and variable clinical progression is consistent with the fact that these diseases necessitate different treatments [10]. However, most of the current treatments for ATLL fail to induce long-term remission and do not offer the prospect of a cure. Even the clinically less aggressive forms of ATLL eventually progress to the acute form. The 4-year survival rate for acute, lymphoma, chronic, and smoldering type ATLL is 5.0, 5.7, 26.9, and 62.8%, respectively [11, 12]. The poor prognosis of ATLL patients is associated with the resistance of neoplastic cells to the conventional combination of high-dose chemotherapy and radiotherapy. While most HTLV-I-infected individuals remain asymptomatic carriers, 1 to 5% of infected individuals will develop ATLL in their lifetime. The disease usually develops after a long latency of several decades, although faster disease progression has been reported in individuals coinfected with parasites. The low incidence and long latency of HTLV-I-associated ATLL suggest that, in addition to viral infection, accumulations of genetic alterations are required for cellular transformation* in vivo*. These observations are consistent with the fact that HTLV-I does not transduce an oncogene and that the viral oncoprotein Tax has a low transforming activity in human T cells [13, 14]. Although HTLV-I integrates into open transcriptionally active chromatin [15], the provirus does not integrate at specific sites within the human genome and therefore HTLV-I is not associated with insertional mutagenesis by either disruption of tumor suppressor or activation of oncogene. The mechanism by which HTLV-I induces T-cell transformation is still unclear but recent studies suggest that the virus may reprogram infected cells to a mutator phenotype. HTLV-I viral proteins can inflict DNA breaks and simultaneously prevent proper repair through the homologous recombination DNA repair pathway, resulting in the accumulation of mutations and small deletions. If the longevity of infected cells is extended through reactivation of hTERT, then the cumulative risk of acquiring a sufficient number of oncogenic events for transformation is significantly increased. This review will describe how common tumor suppressors frequently inactivated in human cancers are affected in ATLL tumor cells and the significance of these alterations in terms of disease progression and therapeutic opportunities.

## 2. Review

### 2.1. Inactivation of Cell Cycle Checkpoints Leads to Uncontrolled Proliferation of ATLL Cells

#### 2.1.1. p53 and p73

In contrast to oncogenic events, inactivation of tumor suppressor functions requires the loss of both alleles. Consistent with this notion, monoallelic loss of the 17p13.1 region, where the p53 gene is located [16, 17], is consistently associated with mutations of the residual p53 allele to inactivate the remaining p53 function [18, 19]. While p53 mutations are relatively uncommon in non-HTLV-I-associated T-cell neoplasms and found in less than 3% of patients [20], it has been reported in approximately 30% of ATLL patients [21–24]. In addition, functional inactivation of p53 in the absence of genetic mutations has been reported in a majority of ATLL patients [25–27]. Further studies demonstrated that the viral Tax protein plays an active part in this process and inactivates p53 transcriptional functions [28–32]. Since significant Tax mRNA expression is detected in approximately 50% of fresh ATLL patient samples analyzed [33, 34], it is unclear if and how p53 is inactivated in ATLL cells in which Tax is not expressed and in the absence of mutations. MdmX is upregulated in HTLV-I-transformed cells* in vitro* and* in vivo* and may play an important role in the inactivation of p53 in the absence of Tax expression. In addition, while p53 mutations in ALL are very rare, hypermethylation of the p53 promoter can be detected in 30% of ALL patients [35]. Such a mechanism could also take part in ATLL and this warrants additional studies. Interestingly, microRNA miR-150 has been shown to target p53 and to play an important role in NSCLC tumorigenesis [36]. Along these lines, miR-150 expression has been found to be upregulated in ATLL patient samples, suggesting that it may be involved in inhibition of p53 in ATLL cells [37]. Similarly, studies have demonstrated that p53 inactivation involves activation of the canonical NF-kB pathway [38] and activation of NF-kB in the absence of Tax can be achieved in ATLL cells through upregulated expression of miR-31 [39], suggesting that miR-31 may play a role in p53 inactivation. Although p53 is transcriptionally inactive in a majority of ATLL patients, several studies have demonstrated that inactivation mechanisms are reversible and that reactivation of p53 functions can activate the senescence or apoptosis pathway and efficiently eliminate HTLV-I-transformed cells [40, 41]. Coexistence of tumor clones with wild type p53 and p53 mutated has been reported in previously untreated ATLL patients. In this patient treatment leading to the eradication of the p53 wild type tumor clone resulted in disease relapse, the emergence of the p53 mutated tumor clone, and aggressive disease progression [41].

The p53-related gene, p73, is located in a chromosome region (1p36) which is a locus that is frequently deleted in human tumors but infrequently mutated [42, 43]. Existence of p73 as a tumor suppressor is debated. Hypermethylation-associated loss of p73 gene expression in various types of leukemia has been reported [44] and, similarly, p73 is inactivated by methylation in 30% of smoldering ATLL but surprisingly at a much lower rate in chronic and acute types of ATLL, with an overall methylation rate of 10% in ATLL [45]. In addition to epigenetic inactivation, the viral Tax protein has been shown to inhibit p73 functions [46, 47].

#### 2.1.2. CDKN2 Genes

The CDKN2A locus located at chromosome 9p21.3 encodes p14ARF and p16INK4a while the CDKN2B locus encodes p15INK4b, a functional homolog of p16INK4a. The p14ARF and p16INK4a genes have been implicated as tumor suppressor genes and are frequently mutated, deleted, or inactivated through promoter hypermethylation in human cancers [48]. P16INK4a is a cyclin-dependent kinase inhibitor (CDKI) that complexes with CDK4 or CDK6 and prevents the activation of CDK-cyclin D and cell cycle progression from G1 to S phase.

While mutations of p16INK4a have been frequently reported in pancreatic adenocarcinoma and melanoma [49–51], genetic mutations have not been reported in ATLL patients. In contrast, homozygous deletion or promoter hypermethylation of the CDKN2A genes has been described in at least 20% of acute ATLL patients and loss of CDKN2A was infrequent in chronic or smoldering ATLL [52–54]. Consistent with its role as a tumor suppressor, azacitidine-mediated demethylation of the p16INK4a locus significantly increased expression of p16INK4a and inhibited the growth of ATLL cells [55]. Remarkably, most of the patients with CDKN2 gene alterations had the acute and most aggressive form of ATLL, which is consistent with the fact that p16INK4A expression is a biomarker associated with a more favorable prognosis as measured by cancer-specific survival (CSS) and recurrence-free survival (RFS) in many human cancers. In pediatric cases of ATLL (2–18 years old), frequency of deletion of the CDKN2A locus or mutation of p53 was found in five of the eight patients, suggesting that alteration in these genes is associated with a more rapid progression of ATLL [56]. Additional epigenetic control of P16INK4a, P19INK4d, and p14ARF expression by microRNAs miR-31 and miR-24 has previously been observed [57, 58]. However, loss of miR-31 expression has been detected in ATLL cells [59] and expression of miR-24 has not been reported. In addition to genetic or epigenetic control of p16INK4A, several studies have shown that the viral Tax protein directly interacts with p16INK4A and prevents its inhibitory activity towards CDK4 [60, 61]. Tax was also shown to bind to p15INK4b similarly to p16INK4a, but not to p18INK4c and p19INK4d. However, expression of p18INK4c was suppressed by Tax at the transcriptional level through the E-box element present in the p18INK4c promoter [60].

**Figure 1:**
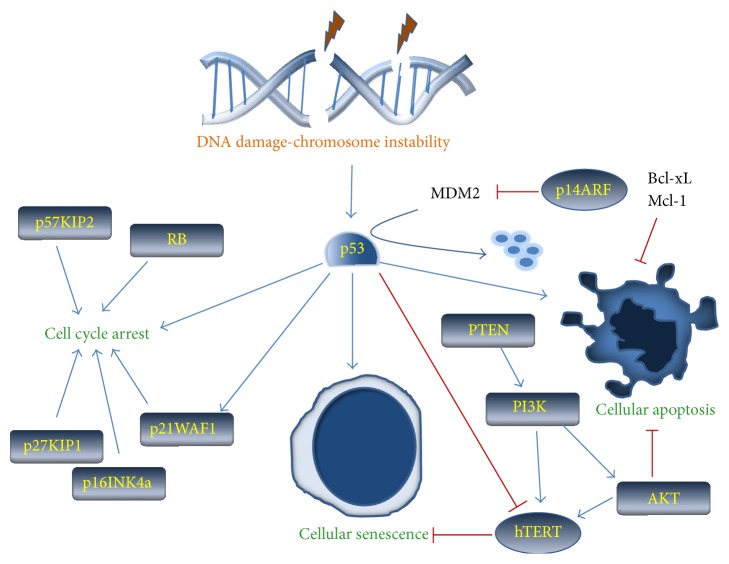
Schematic representation of DNA damage-induced p53 pathway and how other tumor suppressors are connected to activate cell cycle arrest, senescence, or apoptosis to prevent cellular transformation.

P14ARF is encoded from an alternative reading frame of the CDKN2A locus. P14ARF interacts with and sequesters MDM2, thereby preventing the ubiquitination and degradation of p53 (Figure 1). Although P16INK4a and P14ARF act on distinct targets, they share functional similarity by preventing cell cycle progression through inactivation of RB and p53, respectively. However, P14ARF can also inhibit proliferation in cells lacking expression of p53 or p53 and Mdm2 [62]. Like p16INK4A, P14ARF is silenced by promoter hypermethylation or deletion. Although mutations that impair p14ARF functions have been reported in melanoma, colon, pancreatic, and lung cancer [63–65], no data is available for ATLL.

#### 2.1.3. CIP/KIP Family

The CIP/KIP members act as CDKI and have a wider specificity for CDKs than the INK4 members. At low levels p21CIP1/WAF1 and p27KIP1 stimulate the assembly of the CDK4-cyclin D complex, whereas at higher levels p21CIP1/WAF1 and p27KIP1 inhibit the activity of the CDK-cyclin heterodimers [66, 67]. Similarly, at low concentrations p57KIP2 is able to form an active complex with CDK2-cyclin A, whereas at higher levels p57KIP2 prevents the kinase activity of CDK2 [68].

CDKN1A (p21CIP1/WAF1) located at chromosome 6p21.2 is transcriptionally activated in a p53-dependent and independent manner [69], inhibits the activity of cyclin-CDK2, cyclin-CDK1, and cyclin-CDK4/6 complexes, and negatively regulates cell cycle progression from G1 to S [70]. Although expression of p21CIP1/WAF1 has been reported to be increased in HTLV-I-transformed cells* in vitro *[71], additional studies revealed that p21CIP1/WAF1 expression was frequently downregulated through promoter hypermethylation in acute ATLL cells, complete methylation was found in 25% of patients, and partial methylation was found in 70% of ATLL patients [72]. The fact that an increased level of p21WAF1/CIP1 is not associated with cell cycle arrest in HTLV-I-transformed cells* in vitro* may be explained by p21CIP1/WAF1 phosphorylation at Threonine 145 by the PI3K/AKT pathway resulting in cytoplasmic retention and inactive p21CIP1/WAF1 [72]. Additional regulation of p21CIP1/WAF1 by microRNA miR-93 has been described [73]. Since miR-93 is upregulated in ATLL cells it may play a role in tumor cell proliferation by reducing the p21CIP1/WAF1 level [74].

CDKN1B (p27KIP1) is located at chromosome 12p13.1 and its loss has been proposed to play an essential role in T-cell transformation following HTLV-I infection [75]. P27KIP1 is rarely mutated or deleted in ATLL. Homozygous deletions of p27KIP1 and expression of a truncated nonfunctional p27KIP1 protein (amino acid 76^*∗*^) have been reported in two cases of lymphoma ATLL [76]. In contrast, P27KIP1 expression was downregulated at the posttranscriptional level in HTLV-I-transformed cells* in vitro *[75]. In addition, in cells with physiological levels of p27KIP1, AKT-mediated phosphorylation of p27KIP1 at amino acid residue Threonine 157 resulted in its cytoplasmic localization and inactivity in ATLL cells [72].

CDKN1C (p57, KIP2) is located in the telomeric end of chromosome 11 at the 11p15 locus, which contains several imprinted genes. In addition to its role in the G1-to-S transition, p57KIP2 also contributes to the M-to-G1 transition through activation by p73 [77]. Mutations of CDKN1C are associated with sporadic cancers and with Beckwith-Wiedemann syndrome, a disease characterized by an increased risk of tumor formation in childhood [78, 79]. Loss of heterozygosity (LOH) of p57KIP2 is frequently observed in human cancers [80]. Expression of p57KIP2 is transcriptionally regulated through distinct epigenetic mechanisms. Histone methyltransferase EZH2 has also been shown to suppress p57KIP2 expression through histone H3 lysine 27 trimethylation (H3K27me3) [81] and p57KIP2 is methylated in nearly 50% of newly diagnosed ALL patients [82]. In addition, several microRNAs have also been shown to downregulate p57KIP2 expression, including miR-221 and miR-222 [73, 83, 84], miR-25 [73], and miR-92b [85]. However, there is currently no information about the relative expression of these microRNAs in ATLL patient samples and expression of p57KIP2 in ATLL has not been reported.

#### 2.1.4. Retinoblastoma (RB)

The RB locus is located at chromosome 13q14.1. In resting cells inhibition of the cyclin D-CDK4/6 complexes maintains RB and its related proteins (p107 and RB2/p130) in a hypophosphorylated state and sequesters the E2F transcription factor [86]. Following activation of the cyclin D-CDK4/6 complexes, RB becomes hyperphosphorylated and dissociates from E2F, allowing the latter to stimulate expression of the genes involved in the progression to S phase of the cell cycle. RB is progressively dephosphorylated during the M-to-G1 transition [87]. Genetic mutation in the RB gene has been reported in various human cancers [88].

Homozygous loss of RB exon 1 has been reported in one of 21 acute ATLL, one of 15 chronic ATLL, and none of four lymphoma ATLL samples. In that study no point mutations were found in the entire RB gene coding sequence [89]. Hence, the overall rate of RB alteration in ATLL is approximately 5%. Interestingly, the authors observed that none of the samples with an altered RB gene had any defect in CDKI genes and vice versa, suggesting that these tumor suppressor genes likely operate in a common pathway and alteration of either can provide these cells with a growth advantage [89]. These results are consistent with another study suggesting that RB is infrequently mutated or deleted in ATLL tumor cells [22]. In addition, mutations of the RB2/p130 gene have been found in approximately 2.5% of ATLL patients [90, 91]. Despite a low level of genetic alterations in the RB gene in ATLL, almost 50% of patients demonstrate very low levels of expression of an RB protein, which has led to the hypothesis that RB is posttranscriptionally downregulated in ATLL cells [91]. This observation is clinically relevant since lower pRB levels in ATLL patients have been correlated with poor prognosis and shorter survival [92]. Epigenetic control by microRNA may in part explain lower levels of RB protein in the absence of decreased RNA levels. Along these lines, several studies have shown that RB is targeted by several microRNAs, including miR-155 [93], and since miR-155 is highly expressed in ATLL cells [37], this may explain the partial loss of RB. Finally, as indicated above for other tumor suppressors, the viral Tax protein is also able to inactivate RB. Direct binding of Tax to RB targeted the latter for proteasomal degradation and stimulation of cell cycle progression [94].

## 3. Phosphatase and Tensin Homolog (PTEN)/Src Homology 2 Domain Containing Inositol

### 3.1. Polyphosphate Phosphatase (SHP1/PTPN6)

PTEN is located at chromosome 10q23.3 [95] and is among the most frequent tumor suppressors lost in human cancers [96]. PTEN acts as a phosphatase to deactivate phosphatidylinositol (3,4,5)-trisphosphate (PIP3) and inhibit the activation of the PI3K/AKT prosurvival pathway [97]. Germline PTEN mutations and sporadic mutations have been reported in various human cancers [98] but to date no study has investigated the presence of mutations or methylation of the PTEN promoter in ATLL cells. Although PTEN is downregulated at the protein level, in a majority of IL-2-independent HTLV-I-transformed cells* in vitro*, PTEN protein expression is not altered in ATLL tumor cells [99].

SHP1 is located on chromosome 12p13, a region commonly involved in leukemia-associated chromosomal abnormalities. Like PTEN, SHP1 is also implicated in the degradation of PIP3 and inhibition of the PI3K/AKT pathway [100, 101]. In addition, loss of SHP1 enhances JAK3/STAT3 signaling and decreases proteasome degradation of JAK3. Hypermethylation of the SHP1 promoter is associated with loss of SHP1 expression [102, 103], which coincides with the IL-2-independent transformation of T cells by HTLV-I* in vitro *[104, 105]. Consistent with these observations, SHP1 is one of the most frequently altered genes in ATLL patients, with an overall hypermethylation rate of 90% [45]. Interestingly, methylation inactivation of the SHP1 promoter was more frequently seen in the acute (60%) and the lymphoma (80%) form of ATLL, which have the worst prognosis and lowest survival rate [45]. Additional epigenetic control of PTEN and SHP1 has been reported, such as miR-221, miR-222 [106], miR-21 [107], and miR-155 [108]. Among these microRNAs, miR-155 is the only one that has been shown to be upregulated in HTLV-I-transformed T cells* in vitro* as well as in ATLL cells [37].

### 3.2. Secreted Frizzled-Related Protein 1 (SFRP1)

SFRP1 is located at chromosome 8 p12, a region frequently deleted in human cancers [109, 110]. SFRP1 functions as a tumor suppressor and its expression is lost in many patient tumors because of promoter hypermethylation [109, 111, 112]. Point mutation is not a frequent method of inactivation of the SFRP1 gene in cancer [109]. Inactivation of SFRP1 is associated with constitutive Wnt signaling and an increased proliferation in tumor cells [113]. Although potential alterations in SFRP1 have not yet been investigated in HTLV-I-transformed cells, it has been found that the noncanonical Wnt pathway is activated and Wnt5a overexpressed in ATLL cells.

### 3.3. Additional Tumor Suppressor Pathways

In addition to tumor suppressors involved in cell cycle control and proliferation, cancer is frequently associated with inactivation of tumor suppressors involved in other cellular pathways. Among those, activation of senescence or apoptosis limits survival and prevents the growth of pretumoral cells. Other tumor suppressors are responsible for activation of DNA damage responses and DNA repair pathways, and their inactivation increases the cumulative risk of oncogenic genetic and genomic alterations. Finally, some tumor suppressor genes are involved in the control of adhesion molecules and their loss leads to increased metastasis.

### 3.4. Senescence and Apoptosis

In nontumoral cells the progressive shortening of the telomeres after each cell division limits the proliferative potential by activating the senescence program and permanent cell cycle arrest. Consequently, senescence impedes the accumulation of mutations and genomic defects that are needed for initiation and progression of cellular transformation. In cancer cells, preservation of sufficient telomere ends is ensured by reactivation of human telomerase endogenous reverse transcriptase (hTERT). Initiation of senescence is regulated by the p16INK4a/RB-dependent pathway and a p53-dependent DNA damage response (DDR) pathway. As discussed above, most cancer cells avoid senescence by disruption of tumor suppressor genes p53 and p16INK4a and reactivation of hTERT. Like most other human cancers, reactivation of hTERT expression and activity is found in HTLV-I-associated leukemia and required for long-term proliferation of tumor cells [114]. Some studies reported a correlation between telomerase activity and the progression of ATLL [115, 116]. Apoptosis is a tumor suppressor mechanism that is used to eliminate precancerous cells. HTLV-I-transformed cells and ATLL cells are highly resistant to multiple proapoptotic stimuli, including death receptor-mediated and DNA damage-induced agents compared to normal cells. One major player of ATLL cell resistance to apoptosis is the activation of the NF-kB pathway which controls transcription of numerous antiapoptotic proteins such as Bcl-xL and Mcl-1 or inhibitor of apoptosis (IAP) overexpressed in ATLL cells [117–120]. Additional epigenetic alterations have been reported in ATLL. For instance, hypermethylation of the death-associated protein kinase (DAPK) promoter was identified as one of the contributing factors for the progression of the asymptomatic carrier or smoldering type of ATLL to the acute or lymphoma type ATLL and was altered in 55% of patients [45]. MicroRNAs miR-132 and miR-125a are downregulated in ATLL cells [37]. These microRNAs are downregulated through promoter-mediated methylation in various cancers [121, 122]. miR-132 has been shown to target prosurvival proteins in solid tumors while loss of miR-125 protects myeloid leukemia cells from apoptosis [121, 122]. Overall, alteration in the ability of tumor cells to activate the senescence or apoptosis pathway increases resistance to therapies and is associated with a poor prognosis.

## 4. DNA Repair Pathways and Genome Instability

Genome instability is a hallmark of tumor cells and is involved in the transformation process. ATLL has numerous structural and numerical genomic alterations. Although there is not a specific chromosome alteration typifying ATLL, some genomic defects such as translocations involving 14q32 (28%) or 14q11 (14%) and deletion of 6q (23%) occur more frequently and may play a role in disease progression [123, 124]. Alterations of the DDR and the DNA repair pathways are intimately linked to genome and chromosome integrity. HTLV-I Tax protein inhibits DDR and predisposes to accumulation of genomic mutations [125–127]. Although aneuploidy is frequently observed in HTLV-I-transformed and ATLL cells, it is not linked to defects in the mitotic spindle checkpoint, which is fully functional in these cells [128]. In contrast, centrosome amplification-associated aneuploidy has been reported in ATLL cells and may be involved in chromosome instability and tumor progression [129]. Alternatively, defects in the homologous recombination (HR) pathway observed in Tax-expressing cells [130, 131] may also be responsible for chromosome instability and increased aneuploidy [132].

### 4.1. Single-Strand DNA Damage Repair Pathways

Microsatellite instability (MSI) has been linked to a defect of the DNA mismatch repair (MMR) pathway and has been implicated in a wide array of human cancers. The incidence of MSI in ATLL is significantly higher than in other hematological diseases, suggesting that MSI is a feature of ATLL and may be involved in the progression of the disease [133, 134]. In fact, the hMSH2 gene that contributes to DNA mismatch repair demonstrated two types of polymorphisms (CTT to TTT resulting in Leu changing to Phe at codon 390 in exon 7 and CAG to AAG resulting in Gin changing to Arg at codon 419 in exon 7) in ATLL cells [135]. Additional studies demonstrated loss of MMR-related genes in ATLL cells [136]. HTLV-I viral protein Tax-mediated increased expression of proliferating cell nuclear antigen (PCNA) has been shown to inhibit the activity of the nucleotide excision repair (NER) pathway [137, 138]. Similarly, Tax was also shown to inhibit the base excision repair (BER) pathway [139].

### 4.2. Double-Strand DNA Damage Repair Pathway

Tax has been shown to associate with the minichromosome maintenance MCM2-7 helicase and stimulate premature S phase progression leading to genomic lesions [140]. Follow-up studies confirmed these results and demonstrated that Tax acts as an inducer of genomic DNA double-strand breaks (DDSB) during DNA replication by blocking progression of the replication fork [130, 131]. Importantly, Tax-mediated NF-kB activation prevented DDSB repair by homologous recombination (HR) and increased usage of the error-prone nonhomologous end joining (NHEJ) repair pathway [130, 131]. In addition, reduced expression of human translesion synthesis (TLS) DNA polymerases Pol-H and Pol-K in HTLV-I-transformed T cells and ATLL cells was associated with an increase in DNA breaks at particular genomic regions, such as the c-Myc and the Bcl-2 major breakpoints [130].

## 5. Loss of Adhesion Molecules Stimulates Metastasis of ATLL Cells

Studies have reported that the tumor suppressor in lung cancer 1 (TSLC1/IgSF4/CADM1) is overexpressed in the acute type of ATLL [141]. In addition, others had shown that expression of TSLC1 plays an important role in the organ infiltration of ATLL cells [142]. High levels of intracellular and serum levels of MMP-9 have been reported in ATLL patients and correlated with organ involvement, suggesting that overexpression of MMP-9 in ATLL cells may be in part responsible for their invasiveness potential [143]. Likewise, analyses of histopathological tissue sections from ATLL patients with skin infiltrations revealed increased expression of MMP-2 in fibroblasts surrounding infiltrating ATLL cells, but not in fibroblast biopsies from nondiseased areas. Emmprin was found to be overexpressed and it facilitates MMP-2 production via interactions with fibroblasts, thereby facilitating stromal invasion by tumor cells [144]. Additional studies found that the CADM1 protein is overexpressed in ATLL cells. CADM1 interacted with T-lymphoma invasion and metastasis 1 (Tiam1) leading to Rac activation and stimulated infiltration of tumor cells into various organs [145].
